# The Caries-Arrest Effectiveness of Silver Diamine Fluoride Treatment with Different Post-Treatment Instructions in Preschool Children: A Study Protocol for a Randomized Controlled Trial

**DOI:** 10.3390/dj11060145

**Published:** 2023-06-05

**Authors:** Ivy Guofang Sun, Duangporn Duangthip, Edward Chin Man Lo, Chun Hung Chu

**Affiliations:** Faculty of Dentistry, The University of Hong Kong, Hong Kong SAR, China

**Keywords:** silver diamine fluoride, post-treatment instruction, randomized clinical trial, children, dental caries, early childhood caries

## Abstract

In this 12-month randomized active-controlled clinical trial, we compare two post-treatment instructions for silver diamine fluoride (SDF) therapy in arresting dentine caries. The trial will include at least 254 kindergarten children with active dentine caries. The children will be randomized into two groups and receive a 38% SDF solution applied topically to their carious lesions. Children in Group A will rinse immediately, whereas those in Group B will refrain from rinsing, eating, and drinking for 30 min. One trained examiner will perform the dental examination at baseline and every six months. The primary outcome measurement will be the proportion of caries lesions that become arrested at the 12-month examination. Potential confounding factors and parents’ satisfaction with SDF therapy at baseline and after 12 months will be collected using parental questionnaires. This trial will provide evidence-based information for clinical practitioners to give post-treatment instructions for SDF therapy. This study is registered at ClinicalTrial.gov (USA) (registration number: NCT05655286).

## 1. Introduction

Early childhood caries (ECC) is defined as the presence of one or more primary teeth with decayed, missing (due to caries), or filled surfaces in a child under the age of six (71 months) [1]. A review based on 88 countries shows that the mean ECC prevalence for children aged 3–5 years is 57% [2]. ECC is more common in children from low-income families [3] who cannot afford the restorative dental treatment or do not have access to dental care [4]. According to the global burden of dental disease in 2019, 62.9 million children had decayed teeth, which was attributed to global sociodemographic inequality [5].

In Hong Kong, almost half (46%) of the children aged 3–5 years suffer from ECC, and most of the decayed teeth (95%) remain unrestored [3]. When untreated ECC continues to progress, it can lead to dental pain and systemic infection, negatively affecting children’s quality of life [6]. Currently, the Hong Kong government mainly implements the oral health promotion program by delivering free oral health education materials to kindergarten teachers and parents to manage ECC. No government-subsidized treatment program was provided for young children to treat ECC.

Managing ECC through low-cost and minimally invasive methods is vital to relieve the huge global burden of untreated ECC. Nowadays, silver diamine fluoride (SDF) is regarded as a viable therapy alternative for arresting dentine caries due to its effectiveness, affordability, and accessibility [7]. SDF is available commercially for dental use at various concentrations in some counties [8], including 3.8%, 10%, 12%, 30%, 38%, etc. [9]. SDF is generally used for ECC treatment at 38%, which contains 253,870 ppm silver and 44,800 ppm fluoride ions.

Laboratory studies have confirmed the mechanism of SDF for caries control, including silver’s antibacterial effect, fluoride’s remineralization effect, and the ability to inhibit dentine collagen degradation [9]. A clinical trial on SDF for caries arrest in young children showed no serious adverse events [10]. A systematic review showed that SDF could arrest approximately 80% of treated lesions [11]. In addition, SDF therapy is cost-effective, with the material costing less than one USD per application, making it affordable for children from low socioeconomic families [7,12]. Because SDF treatment produces minimal aerosols, it is considered an alternative to caries management during and after the COVID-19 era [8].

Although SDF is effective in arresting ECC, it has some drawbacks, including a metallic or bitter taste. Some children commented that SDF’s taste and smell are unfavorable and unacceptable. They want to rinse or spit out the excessive SDF after treatment if allowed. The published clinical protocol for the UCSF (University of California, San Francisco) dental clinics stated that clinicians should rinse the treated carious lesion with water after SDF treatment [13]. However, the in vitro results of micro-computed tomography indicated that water rinsing could reduce the radiopacity of SDF-treated decayed teeth, possibly due to the reduced SDF precipitation on the decayed teeth [14].

There is no post-treatment instruction available in the clinical recommendations for SDF by the American Academy of Pediatric Dentistry (AAPD) [15]. We searched the published clinical trials on SDF therapy in PubMed. Some publications mention that children refrained from eating and drinking for 30 min [13,16] or 60 min [17,18] after SDF therapy. Some studies do not mention the post-treatment instructions for SDF treatment [19,20], whereas a few studies state that the SDF-treated lesions were rinsed immediately [21,22]. There is no published study in PubMed investigating SDF’s caries-arrest effectiveness with various post-treatment protocols.

Recently, the World Health Organization (WHO) Collaborating Centre expanded the WHO list of essential medicines and added SDF to a new category in 2021 [12]. Therefore, the evidence-based clinical guideline for SDF treatment is crucial for dental practitioners, dental educators, and dental public health workers. In this study, we aim to compare the effectiveness of the various post-treatment instructions for SDF application in arresting dental caries. The proposed study’s results will provide scientific-based evidence regarding the post-treatment SDF protocol.

## 2. Methods/Design

This study is a prospective, parallel, blind, randomized controlled trial with two arms, following the SPIRIT (Standard Protocol Items: Recommendations for Interventional Trials) [23].

### 2.1. Objective

The objective of this randomized clinical study is to compare the effectiveness of the two post-treatment instructions after application of 38% SDF every 6 months over a 12-month period in arresting dental caries among preschool children.

### 2.2. Hypothesis

The null hypothesis of this study is that there is no difference in the caries-arrest effectiveness of the two post-treatment instructions after application of 38% SDF every 6 months at the 12-month follow-up.

### 2.3. Setting

**Figure 1:**
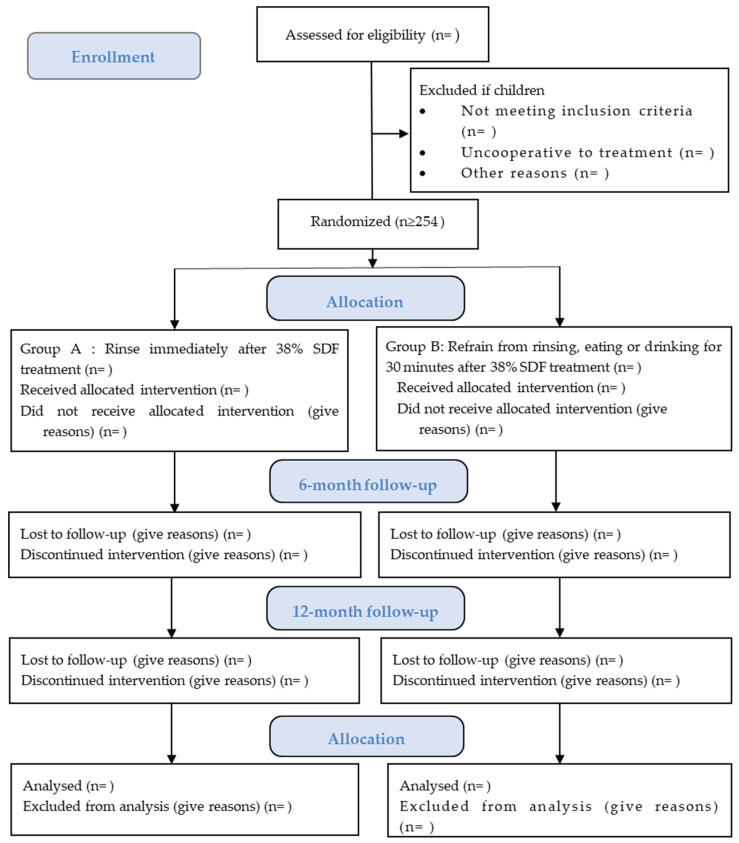
The CONSORT flow diagram.

This clinical trial will be implemented in kindergartens in Hong Kong, where the fluoride concentration in the water supply has been 0.5 ppm since 1988. An invitation letter will be issued to the kindergarten principals to explain this trial’s objective and procedures. After receiving confirmation of participation from the principals, the children’s parents will receive an information sheet and consent through the kindergartens (Supplemental Material S1). In the information sheet, information about the study’s aim, follow-up time, anticipated sample size, the potential benefit and the common side effects of the SDF treatment, the allocation of treatment groups, and the expected treatment outcome will be included. Only the children whose parents read the information sheet and signed the parental consent form will be involved in the dental examination and SDF treatment. Figure 1 shows the CONSORT flow diagram.

The timeline of the study enrolment, intervention, and assessment is shown in Table 1.

### 2.4. Participants

Children in kindergartens will be invited to take part in this clinical trial. The inclusion criteria will be children who (1) have written parental consent, (2) are generally healthy, and (3) have at least one soft dentine caries according to the WHO criteria. Children who (1) are unwilling to accept oral examination or treatment, (2) have a significant systemic disease or take long-term medication that may affect oral health such as children with autoimmune disease, etc., or (3) have developmental dental anomalies such as amelogenesis imperfecta are excluded. Teeth with signs or symptoms of irreversible pulpitis or non-vitality will be excluded. Caries surfaces that were treated with a crown or restoration or arrested will also be excluded.

### 2.5. Recruitment and Examination

#### 2.5.1. Recruitment

A dentist (I.G.S) will be trained and calibrated by an expert (D.D) before the trial. The dentist will screen all children with parental consent in the participating kindergartens. The study children will be instructed to lie on a table and be clinically investigated. The dental examination will be conducted using visual inspection combined with tactile inspection. The visual inspection will be conducted with the aid of a disposable dental mirror mounted on the handle, which is equipped with intra-oral light-emitting diode lighting (MirrorLite, Kudos Crown Limited, Hong Kong SAR, China), and the tactile inspection will be performed using a 0.5 mm WHO Community Periodontal Index (CPI) ball-ended probe (405/WHO probe, Otto Leibinger, Muhlheim, Germany). No dental radiograph examination will be conducted. The children’s dental caries and oral hygiene status will be recorded.

#### 2.5.2. Assessment of Tooth Status

The WHO-recommended criteria for caries diagnosis will be adopted [24], and the number of decayed, missing (due to caries), and filled tooth surfaces (dmfs) will be recorded. Five surfaces of each posterior tooth and four surfaces of each anterior tooth will be thoroughly checked. Additional information, including discoloration, hypermobility, missing not due to caries, and presence of an abscess, will also be recorded.

The information about the recruited carious tooth surfaces will be recorded at baseline and at the time of each follow-up as follows: (1) caries activity status (active/arrested), (2) extent of caries lesion (ICDAS codes 5 and 6), and (3) presence of dental plaque (yes/no). The tooth surface will be diagnosed as active if any softened area on the caries lesion is detected through probing. When the entire caries lesion is detected as hard via probing, it will be recorded as arrested caries. The extent of dentine caries lesions will be assessed following ICDAS criteria [25], using codes 5 (distinct cavities) and 6 (extensive cavities). For those with filled or non-vital teeth at the follow-up, the carious lesions will be regarded as ‘failure’ or ‘active’. Table 2 shows the codes for the assessment of carious lesions (tooth surface level).

#### 2.5.3. Recording oral Hygiene

The visible plaque index (VPI) will be adopted for assessing children’s oral hygiene. The six index teeth’s buccal and lingual surfaces (55, 51, 63, 71, 75, and 83) will be assessed, and the presence of visible plaque (yes/no) will be recorded. The same examiner will conduct the dental examination using the same methods and equipment at the baseline visit and at each follow-up visit. The VPI value of each child will be calculated as a percentage of the total number of tooth surfaces with dental plaque to the total number of tooth surfaces checked.

$$\mathrm{VPI}\;\mathrm{value}=\frac{\mathrm{number}\;\mathrm{of}\;\mathrm{tooth}\;\mathrm{surfaces}\;\mathrm{with}\;\mathrm{dental}\;\mathrm{plaque}}{\begin{array}{c} \mathrm{number}\;\mathrm{of}\;\mathrm{tooth}\;\mathrm{surfaces}\;\mathrm{with}\;\mathrm{dental}\;\mathrm{palque}\\ +\mathrm{number}\;\mathrm{of}\;\mathrm{tooth}\;\mathrm{surfaces}\;\mathrm{without}\;\mathrm{dental}\;\mathrm{palque} \end{array}}\times 100\%$$

Regarding the dental caries and oral hygiene assessment, duplicate examinations on approximately 10% of the recruited children will be conducted to assess the examiner’s intra-reliability at each visit.

### 2.6. Questionnaire Survey

A parental questionnaire consisting of three parts (the child’s basic information, dietary and oral hygiene habits, and family demographic characteristics) will be administered at the baseline and final follow-up visits. It will be used to collect information, including daily tooth brushing, frequency of snack intake, whether parent-assisted tooth brushing is used, whether a child is bottle-fed before sleeping without tooth brushing or mouth rinsing, use of fluoride toothpaste, parental satisfaction with the child’s oral health and appearance, the child’s main caretaker, parental education level, and monthly family income. For the 12-month follow-up period, information on children’s dental treatment experiences will also be included. In addition, a questionnaire about adverse effects, such as systemic toxicity, vomiting, and hospitalization, will be collected after SDF treatment. The grouping of the categorical variables of the questionnaire at baseline and at the 12-month follow-up are summarized in Table 3.

### 2.7. Randomized Allocation, Concealment, and Blinding

This trial will use the stratified randomization method to allocate children randomly into two intervention groups. The recruited children will be firstly distributed into two strata based on their dental caries experiences to achieve balance in the disease severity among the two groups. Children with one to three caries surfaces will be stratified into low-caries group. Children with four or more caries surfaces will be stratified into high-caries group. Then, children in each stratum will be randomly allocated into one of the two treatment groups based on the random number with a block size of six generated by the Excel software.

An assistant who will not participate in the dental examination or treatment intervention will generate the allocation sequence. After the children receive the SDF treatment, the assistant will then open the concealed label to identify the group allocation. The participants know their treatment interventions, but the examiners are blinded and do not know the treatment allocation.

### 2.8. Interventions

All the tooth surfaces with carious lesions will be treated with 38% SDF (Advantage Arrest Elevate Oral Care, FL, USA) solutions. The SDF application will be adopted from the recommended clinical procedures [26] as follows: remove food debris, isolate the decayed tooth with a gauze or cotton roll, and apply SDF with a microbrush on the decayed tooth for 60 s. After SDF treatment, one assistant who helps teachers instructs children to comply with various post-treatment protocols will allocate children to one of the two intervention groups. The dentist will not know the group allocation. The following are the two post-treatment protocols. Children in Group A will be instructed to rinse their teeth with a cup of about 50 mL of water immediately after SDF treatment. Afterward, no post-treatment instruction is given to the children. Children in Group B will be instructed not to rinse, eat, or drink for 30 min.

A research assistant will record and monitor the children’s compliance with the intervention in each group at each treatment visit. The intervention will be conducted at baseline and at the 6-month follow-up. All examinations and interventions will be conducted in school settings.

### 2.9. Outcome Measure

#### 2.9.1. Primary Outcome

The primary outcome measurement will be the proportion of caries lesions that become arrested at the 12-month examination.

#### 2.9.2. Secondary Outcome

Parental satisfaction about their child’s teeth appearance and oral health status will be evaluated prior to the baseline examination and at the 12-month examination. The SDF treatment’s adverse effects will also be assessed after SDF application.

### 2.10. Sample Size Calculation

According to the published studies, approximately 70% of active dentine caries in primary teeth were halted after 38% SDF treatment [27]. Clinical significance would be defined as an absolute difference of 10% in the arrested caries rate between the groups. A two-tailed test with a statistical significance of 0.05 (α = 0.05) and a power of 80% (β = 0.2) will be set. The Sealed Envelope website will be used to calculate the sample size (https://www.sealedenvelope.com/, accessed on 1 November 2022). At least 708 active carious tooth surfaces in total (354 per group) are needed.

The anticipated intraclass correlation coefficient for the caries data at the tooth surface level within a child is 0.13. Following the results of the published study, the mean number of decayed tooth surfaces per child was 5 at baseline. According to the sample size calculation for a multilevel statistical model, the estimated design effect is 1.52. Hence, at least 1077 decayed tooth surfaces are needed. After 12 months, the anticipated dropout rate is approximately 15%. Therefore, at least 1267 tooth surfaces and 254 children in total (127 per treatment group) need to be recruited at baseline.

### 2.11. Data Analysis

An assistant will enter the collected data into the Excel file and then proofread the data entry. The proofread data will be imputed to IBM’s SPSS 26.0 software (IRB Corp., Armonk, NY, USA) for statistical analysis. Cohen’s Kappa test will be employed to test the intra-examiner agreement for caries assessment. A statistical significance level will be set at 0.05 for all tests.

Chi-square (χ^2^) test will be conducted to assess the differences between the two intervention groups regarding their demographic background (sex, birthplace, family monthly income, parental education levels), their baseline clinical features (caries extent, tooth type, presence of dental plaque on decayed tooth surfaces), and their oral health-related habits (use of fluoride toothpaste and snacking). The baseline differences in VPI value and dmfs value between the two groups will be analyzed using a *t*-test. A logistic regression model will be used to assess the associations between the outcomes and other potential factors.

Regarding the clustering effect, because one child may have more than one untreated carious tooth surface, the lesions in the same child may be correlated. Therefore, two-level (subject level and tooth surface level) generalized estimating equation models will be used to assess the independent variables associated with the effectiveness of post-treatment protocols in arrested caries after 12 months. The independent variables include the children’s demographic information (sex, birthplace, family monthly income, parental education levels), their baseline clinical features (caries extent, tooth type, presence of dental plaque on decayed tooth surfaces, caries experience, and oral hygiene status), and children’s oral health-related behaviors (use of fluoride toothpaste, type of bottle feeding, and snacking habits). The McNemar test will be used to investigate changes in parental satisfaction regarding their child’s oral health and appearance at baseline and at the 12-month examinations.

### 2.12. Ethical Considerations and Registration

Ethics approval was obtained from the Institutional Review Board of the University of Hong Kong/Hospital Authority Hong Kong West Cluster (UW 22-667). After each examination, the oral examination report will be sent to the child’s parents to report the number of untreated dental caries, whether to receive the SDF treatment, and the suggestions of the examiners for the children’s dental treatment needs, such as extraction of the retained root and pulp treatment. Parents could bring their children to the hospital for their children’s teeth at their expense. Medical advice will be given to parents if there are any adverse effects related to the SDF treatment. This clinical trial was registered at ClinicalTrials.gov under the registration number NCT05655286 in December 2022. The recruitment process for participation started in February 2023. The recruitment process is scheduled to end on 31 June 2023.

## 3. Discussion

SDF treatment is a simple, affordable, and effective treatment for caries control in children. The US Food and Drug Administration (FDA), the AAPD, and the WHO have regarded it as an essential strategy for oral health management to address the burden of dental caries [12,28,29]. Recently, SDF treatment has been adopted in community-based dental programs to manage ECC in many countries or regions, including Argentina, Australia, Brazil, Hong Kong in China, Egypt, Finland, India, Japan, Mongolia, South Africa, the United Kingdom, the United States, etc. However, a review shows that the guidelines for SDF vary significantly in different countries, and there is still no agreement between clinicians regarding the SDF treatment protocol due to limited supporting evidence [8].

A study shows that SDF therapy is a child-friendly method to treat ECC in the kindergarten setting [30]. In Hong Kong, a territory-wide dental outreach project using SDF for caries control has been implemented in 1000 kindergartens (https://www.jccohp.hku.hk, accessed on 1 November 2022) since 2020. This project was funded by a non-governmental organization and performed by the Faculty of Dentistry at the University of Hong Kong, aiming to improve the oral health of kindergarten children. The children are instructed not to eat or drink for 30 min after receiving the SDF treatment. The 30 min are arbitrarily set in order to allow more contact time between SDF and the carious lesions. It is supposed that the prolonged contact time enhances the effectiveness of SDF to arrest caries, but the evidence is lacking.

The instruction of no eating or drinking for 30 min requires teachers to monitor the SDF-treated children. This is difficult to implement when the service time is near lunch or break time. Moreover, the instruction sometimes disturbs the school schedule. In addition, SDF’s unpleasant taste may be alleviated if no restriction about refraining from rinsing, drinking, and eating is imposed. A lack of post-treatment instruction may be desirable and practical if the caries-arrest effectiveness is not diminished. However, questions remain about whether a lack of post-treatment instruction may deteriorate SDF’s caries-arrest efficacy. The results of this clinical trial will give an evidence-based suggestion for post-treatment instruction in SDF therapy.

The published clinical trial revealed that 38% SDF is more effective in arresting dental caries in primary teeth than 12% SDF, and the caries lesions with semi-annual SDF application have a higher arrest rate than annual SDF application [27]. Hence, in this clinical trial protocol, 38% SDF with semi-annual application will be adopted to treat ECC. Due to the study being conducted after the COVID-19 pandemic, we have prepared to invite more children to join the study compared to the previous studies, as the participation rate may be lower, and the drop-out rate may be higher than the results of the previous clinical trials conducted in Hong Kong.

We will record the extent of the carious lesions, dental plaque on lesions, the tooth position, and the status of pulp vitality. We will also collect children’s demographic information and oral health-related behaviors. These are potential confounding factors that may influence the study’s primary outcome. Through the analysis of these factors, we expect to provide comprehensive suggestions for SDF post-treatment instructions adjusted for the confounding factors. The in vivo study showed that following the restoration, a remaining dentine thickness (RDT) of 0.5 mm or greater is necessary to avoid pulp injury [31,32]. However, in this study, visual inspection and tactile detection will be used for assessing caries activity. The X-ray examination will not be used, because it is not feasible and safe in the kindergarten setting. Therefore, RDT will not be recorded in this study, and this is one limitation of this study. In addition, it is uncertain whether unintentional daily fluoride intake through fluoridated toothpaste (systemic effect) may affect caries arrest in young children. However, due to the study’s limitations, the assessment of daily fluoride intake could not be measured.

The strength of this study includes a sufficient sample size, which generates adequate study power. This study protocol describes the specific and detailed procedure for conducting a prospective, blinded, randomized clinical trial. Any materials and equipment used are explicitly listed. The protocol of procedures is based on the published peer-reviewed articles, and the method steps are reproducible. A limitation of this trial is that the participating children cannot be blind to the group intervention because they need to perform rinsing or refrain from rinsing according to their treatment allocation. Nevertheless, the awareness of the treatment allocation among preschool children is unlikely to influence the study outcomes. Regarding the external validity, this clinical trial will be conducted in kindergarten children. Therefore, the results may not be translatable to other age groups.

This randomized controlled clinical trial will provide evidence on whether it is necessary to give a post-SDF treatment instruction to refrain from eating and rinsing for 30 min. The results of this study will be beneficial for dental practitioners and dental public health workers in the adoption of SDF for caries control in young children.

## Figures and Tables

**Table 1:** The schedule of enrolment, intervention, and assessments.

	STUDY PERIOD			
	Enrolment	Allocation	Post-Allocation	
TIMEPOINT	*−t*_1_	0	6 m	12 m
ENROLMENT:				
Eligibility screen	X			
Informed consent	X			
Allocation		X		
INTERVENTIONS:				
[Group A]		X	X	
[Group B]		X	X	
ASSESSMENTS:				
[Active caries]	X	X	X	X
[Arrested caries]			X	X

“X” means time point of each action.

**Table 2:** Code for assessment of carious lesions (tooth surface level).

Carious Lesion	Code
Caries activity status	1: active, 2: arrested
Extent of caries lesion	1: distinct cavities, 2: extensive cavities
Presence of dental plaque	0: no, 1: yes
Non-vital signs (at tooth level)	0: no, 1: abscess/fistula, 2: pulpal involvement

**Table 3:** Grouping of categorial variables of the parental questionnaire.

Variable	Group	Data Collection Period
Sex	Male Female	Baseline
Birthplace	Hong Kong Mainland China Other	Baseline
Serious systemic disease	Yes No	Baseline
Long-term medication	Yes No	Baseline
Monthly family income	HKD 20,000 or below HKD 20,001–40,000 HKD 40,001 or above	Baseline
Mother’s education level Father’s education level	Junior secondary school Senior secondary school Post-secondary or university	Baseline
Main caretaker	Parents Grandparents Maid or others	Baseline
Bottle-feeding before bed	Yes No	Baseline and 12-month follow-up
Sweet snack frequency	Less than once Once Twice More than twice	Baseline and 12-month follow-up
Daily tooth brushing	Yes No	Baseline and 12-month follow-up
Use of fluoride toothpaste	Yes No Not sure	Baseline and 12-month follow-up
Parents’ satisfaction for child’s dental appearance	Very satisfied Satisfied Dissatisfied Very dissatisfied	Baseline and 12-month follow-up
Parents’ satisfaction for child’s dental health	Very satisfied Satisfied Dissatisfied Very dissatisfied	Baseline and 12-month follow-up
Dental treatment during the last one year	Yes No	12-month follow-up
Category of the dental treatment	Check-up Fluoride treatment Filling Extraction Others	12-month follow-up

## Data Availability

The results of each follow-up examination of each child will be shared to his/her parents. The interim and results will be presented in international conferences.

## Supplementary Materials

The following supporting information can be downloaded at https://www.mdpi.com/article/10.3390/dj11060145/s1, S1: Parental consent.
